# Supplementary material for: In-silico study of approved drugs as potential inhibitors against 3CLpro and other viral proteins of CoVID-19
Source: PLoS One. 2025 Jun 25;20(6):e0325707. doi: 10.1371/journal.pone.0325707 (PMC12193675; doi:10.1371/journal.pone.0325707)
Supplement: S1 Table — (PDF) [file pone.0325707.s001.pdf]

Table Hydrophobic and H bonding interaction of top ranked ligands with 7JSU, 7LM9 and 7DE1 Proteins.

Protein Name	Zinc ID	AA Hydrophobic	Distance (Å)	AA H Bonding	Distance (Å) H-A D-A	Donor Angle
7JSU	ZINC085432544	PHE140(A)	3.94	ASN142(A)	2.56 3.23	122.86
		MET165(A)	3.60	ASN142(A)	2.48 3.30	136.86
		GLU166(A)	3.06	GLU166(A)	3.01 3.65	121.92
				GLN189(A)	3.45 3.80	102.50
7JSU	ZINC003873365	GLU166(A)	3.22	HIS163(A)	2.36 3.18	136.68
7JSU	ZINC085536956	PHE140(A)	3.89	ASN142(A)	3.09 3.37	140.66
		LEU141(A)	3.95	ASN142(A)	2.51 4.04	155.73
		MET165(A)	3.73	GLU166(A)	2.97 3.61	121.57
		GLU166(A)	3.06	GLN189(A)	3.51 3.86	102.95
7JSU	ZINC261494640	GLU 166(A)	3.92	ASN142(A)	2.93 3.45	113.86
				ASN142(A)	2.91 3.73	138.46
				ASN142(A)	2.36 3.18	139.18
		GLN 189(A)	3.83	GLY143(A)	1.92 2.93	170.34
				GLN189(A)	2.43 3.26	138.41
				ASN142(A)	2.93 3.45	113.86
7JSU	ZINC008214470	PHE140(A)	3.62	ASN142(A)	3.17 3.78	120.04
		GLU166(A)	3.50	GLU166(A)	2.76 3.61	140.74
				GLU166(A)	2.43 3.36	159.55
7LM9	ZINC085432544	GLU327(A)	3.76	ASN142(A)	2.49 3.02	130.61
		VAL354(A)	3.85	GLU166(A)	1.98 2.34	122.12
7LM9	ZINC003873365	PHE360(A)	3.78	SER30(L)	2.48 3.02	162.50
7LM9	ZINC085536956	PHE360(A)	3.55	SER30(L)	2.34 3.28	165.88
				THR359(A)	2.91 3.87	163.72
7LM9	ZINC261494640	LEU322(A)	3.65	ARG96(H)	2.89 3.90	168.39
		ASN330(A)	3.96	VAL98(H)	1.90 2.90	163.25
		VAL354(A)	3.69	THR332(A)	2.40 2.79	103.31

7LM9	ZINC008214470	PHE360(A)	3.66	SER30(L)	2.04 2.70	123.24
				SER31(L)	2.89 3.35	108.99
				TYR32(L)	3.10 3.53	108.93
7DE1	ZINC085432544	LYS261(A)	3.88	LYS261(A)	2.42 3.02	117.10
				THR263(B)	2.01 2.94	161.29
				ALA305(B)	2.77 3.34	117.22
7DE1	ZINC003873365	GLN303(B)	3.92	GLN303(B)	2.03 2.88	142.95
		ILE351(B)	3.67			
		LEU352(B)	3.28			
7DE1	ZINC085536956	LYS261(A)	3.89	LYS261(A)	2.41 3.02	117.26
		ALA305(B)	3.43	THR263(B)	2.00 2.94	162.16
7DE1	ZINC261494640	ASP288(B)	3.27	ASP348(B)	3.57 3.71	140.56
		GLN289(B)	3.68			
		HIS300(B)	3.84			
		ILE351(B)	3.64			
		LEU352(B)	3.41			
		LYS355(B)	3.88			
		ALA359(B)	3.65			
		PHE363(B)	3.79			
7DE1	ZINC008214470	ASP288(B)	3.98	ASN354(B)	2.06 2.90	143.89
		ILE351(B)	3.79			
		ASN354(B)	3.70	THR362(B)	2.83 3.63	141.04
		ALA359(B)	3.94			
