# Supplementary material for: In-silico study of approved drugs as potential inhibitors against 3CLpro and other viral proteins of CoVID-19
Source: PLoS One. 2025 Jun 25;20(6):e0325707. doi: 10.1371/journal.pone.0325707 (PMC12193675; doi:10.1371/journal.pone.0325707)
Supplement: S2 Table — (PDF) [file pone.0325707.s002.pdf]

Table . Description of the top-ranked five drug compounds.

Zinc ID	Molecular Formula	SMILES with Key Structural Features	2D Structure
ZINC085432544	$C_{46}H_{58}N_4O_9$	CC[C@]1(O)C[C@@H]2CN(CCc3c([nH]c4ccccc34)[C@@](C(=O)OC)(c3cc4c(cc3OC)N(C)[C@H]3[C@@](O)(C(=O)OC)[C@H](OC(C)=O)[C@]5(CC)C=CCN6CC[C@]43[C@@H]65)C2)C1<br>Carboxyl Group (-COOH): Bond angles ~120°, C=O bond length ~1.23 Å, C-OH bond ~1.43 Å.<br>Amino Group (-NH2): Bond angle ~109.5°, N-H bond length ~1.01 Å.<br>Ring Systems: bond angles ~120°.	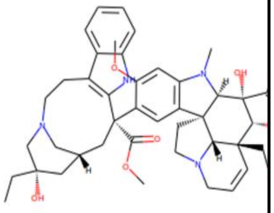
ZINC003873365	$C_{21}H_{32}N_2O$	C[C@]12Cc3cn[nH]c3C[C@@H]1CC[C@H]1[C@@H]2CC[C@]2(C)[C@H]1CC[C@@]2(C)O<br>Chlorine Atoms (Cl): C-Cl bond length: ~1.77 Å, bond angles ~109.5°.<br>Amino Group (-NH2 or -NHR): Bond angles ~109.5°, N-H bond length: ~1.01 Å.<br>Carbonyl Group (C=O): C=O bond length: ~1.23 Å, bond angle ~120°.<br>Aromatic Rings: bond angles ~120°	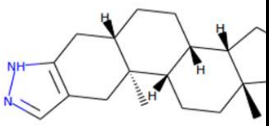

ZINC085536956	C45H54N4O8	CCC1=C[C@H]2CN(C1)Cc1c([nH]c3ccccc13)[C@@](C(=O)OC)(c1cc3c(cc1OC)N(C)[C@H]1[C@@](O)(C(=O)OC)[C@H](OC(C)=O)[C@]4(CC)C=CCN5CC[C@]31[C@@H]54)C2<br><br>Carboxyl Group (-COOR): C=O bond length: ~1.23 Å, C-O bond length: ~1.43 Å.<br>Amino Groups (-NH2): Bond angles ~109.5°, N-H bond length: ~1.01 Å.<br>Long Alkyl Chains: C-C single bond lengths around ~1.54 Å and bond angles of ~109.5°.<br>Ring Structures: bond angles ~120°.	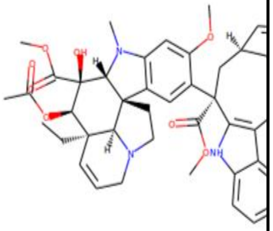
ZINC261494640	C50H77NO13	CO[C@H]1C[C@@H]2CC[C@@H](C)[C@@](O)(O2)C(=O)C(=O)N2CCCC[C@H]2C(=O)O[C@H]([C@H](C)C[C@H]2CC[C@H](O)[C@@H](O)C2)CC(=O)[C@@H](C)/C=C(/C)[C@@H](O)[C@H](OC)C(=O)[C@H](C)C[C@H](C)/C=C\C=C/C=C\1C<br><br>Thio Group (-S): C-S bond length: ~1.82 Å, bond angle ~109.5°.<br>Carbonyl Groups (C=O): C=O bond length: ~1.23 Å, bond angle ~120°.<br>Amino Groups (-NH2): N-H bond length: ~1.01 Å, bond angle ~109.5°.<br>Aromatic Rings: bond angles ~120°	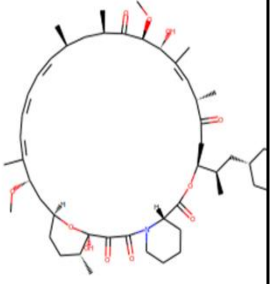

ZINC008214470	C43H55N5O7	CC[C@]1(O)C[C@H]2CN(CCc3c([nH]c4ccccc34)[C@@](C(=O)OC)(c3cc4c(cc3OC)N(C)[C@H]3[C@@](O)(C(N)=O)[C@H](O)[C@]5(CC)C=CCN6CC[C@]43[C@@H]65)C2)C1<br>Carbonyl Groups (C=O): C=O bond length: ~1.23 Å, bond angle ~120°.<br>Amino Group (-NH2 or Heterocyclic N): N-H bond length: ~1.01 Å.<br>Aromatic Rings: bond angles ~120°.	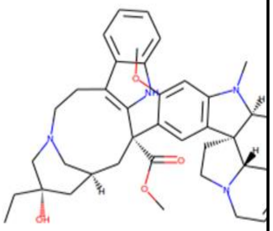
