# Supplementary material for: In-silico study of approved drugs as potential inhibitors against 3CLpro and other viral proteins of CoVID-19
Source: PLoS One. 2025 Jun 25;20(6):e0325707. doi: 10.1371/journal.pone.0325707 (PMC12193675; doi:10.1371/journal.pone.0325707)
Supplement: Attachment — Submitted filename: Review Report_Dr. Aya.docx [file pone.0325707.s003.docx]

Dear Dr. Imra Aqeel *et al.*,

Regarding the manuscript titled (To explore the potential inhibitors against multi-target proteins of CoVID-19 using in-silico study); the following comments should be amended accordingly:

1- The title should be more descriptive or comprehensive title that fully reflects the scope and depth of your study.
2- The abstract is well-written overall, but by tightening up some sentences and providing a bit more detail on the methodology and results, you can enhance its clarity and impact.
3- Try to improve your introduction and compare your results to others like (<https://doi.org/10.2147/DDDT.S354841>).
4- Write *in silico*, *in vitro*, and *in vivo* words in italic.
5- Overall, the article is well-structured and easy to follow. The introduction provides a clear context for the research, and the progression through various sections is logical and cohesive. However, some sections could benefit from clearer transitions. For instance, the section on in silico approaches could have more detailed explanations about the specific algorithms and software used in the studies, which would provide readers with a deeper understanding of the methodologies.
6- Molecular dynamics simulation (for at least 100 ns) for the most active five complexes, compared to the co-crystallized inhibitor of each target receptor should be per formed to confirm the docking results.
7- Some tables (like Tables 7, 8, and 9) should be moved to the Supplementary Data.
8- A graphical abstract is recommended.
9- The article fails to adequately discuss the importance of **experimental validation** following in silico predictions. While in silico techniques are powerful, the review does not highlight how real-world testing (e.g., in vitro or in vivo experiments) is essential to confirm the efficacy and safety of the identified inhibitors. The reliance on computational data alone can be problematic, and without a proper discussion of the need for follow-up experiments, the paper might give the impression that in silico studies are sufficient on their own.
10- Improve the resolution of the 2D structures in table7, so that the chemical structure, such as bond angles and atom labels are clear even when printed or zoomed in.
11- There are some grammatical and typographical error, please revise it.
