# Supplementary material for: In-silico study of approved drugs as potential inhibitors against 3CLpro and other viral proteins of CoVID-19
Source: PLoS One. 2025 Jun 25;20(6):e0325707. doi: 10.1371/journal.pone.0325707 (PMC12193675; doi:10.1371/journal.pone.0325707)
Supplement: Attachment — Submitted filename: Response to Reviewers.docx [file pone.0325707.s005.docx]

PLOS ONE Manuscript ID: PONE-D-25-07472

To explore the potential inhibitors against multi-target proteins of COVID-19 using in-silico study

**Subject:** Response to Reviewers’ Comments on Manuscript [PONE-D-25-07472]

We would like to thank the editor and reviewers for their valuable comments, suggestions, and queries. These comments have helped to improve the quality of the manuscript. The manuscript is revised in the light of useful comments/suggestions. The modifications are highlighted with yellow color and track changes in the revised manuscript (MS). Kindly, below is a point-by-point response to the reviewers’ comments.

**Editor Comments and Responses:**

**Comment 1:** *[*Please ensure that your manuscript meets PLOS ONE's style requirements, including those for file naming.*]*

**Response:** Thank you for your valuable feedback. We ensured it.

**Comment 2:** *[*We note that the grant information you provided in the ‘Funding Information’ and ‘Financial Disclosure’ sections do not match. When you resubmit, please ensure that you provide the correct grant numbers for the awards you received for your study in the ‘Funding Information’ section.*]*

**Response:** Thank you for bringing this to our attention. We apologize for the inconsistency between the ‘Funding Information’ and ‘Financial Disclosure’ sections. Correct Funding Information is:

“The funding of the Princess Nourah bint Abdulrahman University Researchers Supporting Project number (PNURSP2025R513), Princess Nourah bint Abdulrahman University, P.O. Box 84428, Riyadh 11671, Saudi Arabia.”

**Comment 3:** *[*We note that you have provided funding information that is not currently declared in your Funding Statement. However, funding information should not appear in the Acknowledgments section or other areas of your manuscript. We will only publish funding information present in the Funding Statement section of the online submission form.

Please remove any funding-related text from the manuscript and let us know how you would like to update your Funding Statement. Currently, your Funding Statement reads as follows:

“The author(s) received no specific funding for this work.”

Please include your amended statements within your cover letter; we will change the online submission form on your behalf.*]*

**Response:** Thank you for the clarification. We have removed the funding-related text from the manuscript, as advised. Please update our Funding Statement in online submission form as:

“The funding of the Princess Nourah bint Abdulrahman University Researchers Supporting Project number (PNURSP2025R513), Princess Nourah bint Abdulrahman University, P.O. Box 84428, Riyadh 11671, Saudi Arabia.”

**Comment 4:** *[*We note that your Data Availability Statement is currently as follows: [All relevant data are within the manuscript and its Supporting Information files.*]*

**Response:** Thank you for your observation. We confirm that all data generated or analyzed during this study are included within the manuscript and its Supporting Information files. Data used in this study is already available in public repositories <https://zinc20.docking.org>/ and <https://www.rcsb.org/> and these are mentioned in the manuscript.

**Reviewer 1 Comments and Responses:**

**General comments**: *[*The authors provided a computational approach for identifying promising inhibitors, of natural origin, against multiple SARS-CoV-2 biotargets. They adopted combined molecular docking and machine learning regression approaches to identify potential shits. This manuscript is relevant, valuable in the field of drug discovery. However, there are some points should be considered prior publication.*]*

**Response:** Thanks to the reviewer. Now, we have revised MS text in the light of these useful comments:

**Comment 1:** *[*The authors adopted the PDB.file (7JSU) for performing the computational analysis which is deposited in its monomeric state. Typically, the 3CLpro is a homodimer relating to its biological activity (10.1126 / science.abb3405) and so computational study should have been performed in its homodimeric state since it is the active form of the protein. Additionally, a comparative data analysis of each 3CLpro protomer could be provided for gaining greater insights regarding the effect of ligand binding on dimerization since the dimerization state is proximity with the 3CLpro canonical binding site. It is advised that the authors would discuss this, while elaborating on the adequacy of computational screening on the 3CLpro dimer versus monomer states.
https://doi.org/10.1038/s41598-021-88630-9
<https://doi.org/10.1080/07391102.2021.1880481>*]*

**Response:** We appreciate the reviewer’s insightful comment regarding the relevance of the homodimeric form of 3CLpro and its impact on ligand binding. We revised our manuscript as follow:

“In this research, we used the crystal structure of the SARS-CoV-2 main protease (3CLpro) with PDB ID: 7JSU to conduct molecular docking analysis. This structure exists in its monomeric state in the database, but it has an annotation as a biological assembly in the form of a homodimer (Global Stoichiometry: Homo 2-mer – A2), (<https://www.rcsb.org/structure/7JSU>) which shows the enzyme's active form. We chose to use the monomeric form because it was available in high resolution and many previous virtual screening studies had used it. It's worth noting that AutoDock Vina, the docking tool we used in our analysis works on individual binding pockets and doesn't simulate protein–protein interactions, which makes docking on monomeric structures a sound method. Also, the ligand in 7JSU forms a covalent bond with Cys145, which supports the use of the monomeric model to evaluate inhibitory potential. Earlier studies have shown that the active binding site of 3CLpro the catalytic dyad (His41 and Cys145) is within a single protomer, which allows for valid ligand interaction analysis without the immediate need for the dimeric form [30]. Many computational drug discovery efforts [31] [32] have used monomeric 3CLpro to initial screen and identify leads, which backs up the relevance and adequacy of this approach.

However, the processes of dimerization contribute to the functions of the enzyme and might also affect the stability and movement of the active site [33]. Some of the research studies [34] [35] ]indicated that binding a ligand might influence dimerization as well as the activity of the enzyme. To enhance our docking experiment or widen the range of the structural scope we, too, included the dimeric form of the nucleocapsid (N) protein C terminal domain, PDB ID: 7DE1, in the dimer analysis. This structure has also been shown to exhibit homodimeric stoichiometry (A2) alongside the experimental evidence and the PDB biological assembly. The addition of this dimeric structure illustrates our consideration for the oligomeric states and how fundamental these states are in binding dynamics. Therefore, we approached the problem by integrating computations on structures simplified to their monomeric and dimeric representations which are considered where biologically relevant using geometrical reasoning placed in a contextual framework called virtual screening. This in silico stepwise progression of research design described above verges from monomer-based screening into dimer validation balances computational feasibility with biological relevance during the preliminary stages of drug development. ” [Page 9 -10, Section 2.1.4]

**Comment 2:** *[*The co-crystalline ligand (UED; N~2~-[(benzyloxy)carbonyl]-N-{(2S)-1-hydroxy-3-[(3S)-2-oxopyrrolidin-3-yl]propan-2-yl}-L-leucinamide) at the 7JSU PDB.file has been reported as covalent inhibitor for the virus protein. This Michael acceptor inhibitor was used by the authors for developing models that used to screen libraries of reversible acting drugs. It is recommended to validate the obtained models on other PDB.file with reversible non-covalent 3CLpro inhibitor (e.g. PDB ID: 7L0D).*]*

**Response:** We would like to thank the reviewer for their insightful comments. For further clarification regarding covalent and non-covalent inhibitors, we revised the manuscript as follow:

“Using several protein structures, we investigated the binding of possible inhibitors taking into account both covalent and non-covalent interactions in this research. Covalent ligand (UED) was used for docking research with 7JSU, which represents the monomeric form of the SARS-CoV-2 main protease (3CLpro); we also included non-covalent docking to expand the range of our models. Particularly, 7DE1, which represents the C-terminal domain (CTD) of the SARS-CoV-2 nucleocapsid protein, was used to investigate non-covalent interactions in its dimer form. For study of protein-protein interactions and possible non-covalent inhibitor binding at the dimer interface, this structure is pertinent. Furthermore 7LM9, a complex of an antibody fragment (Fab) with the receptor-binding domain (RBD) of the SARS-CoV-2 spike protein. The interactions here are first and foremost non-covalent, including hydrogen bonding, hydrophobic interactions, and electrostatic forces. Together with the 7JSU monomer, these non-covalent protein structures allow a thorough assessment of covalent and non-covalent inhibitors hence ensuring applicability of our computational models for various drug discovery methods including reversible inhibitors and covalent binders.” [Page 10 -11, Section 2.1.4]

**Comment 3:** *[*Within the molecular modelling studies, the authors are advised to provide more details on the identified target topology and pocket description prior to presenting the docking findings*]*

**Response**: Thanks for this suggestion. We have now included the more description of the binding pockets of the three selected proteins:

“The 7LM9 protein binding pocket harbors ACE2-interacting residues like Tyr449, Gln493, and Asn501 that are pivotal in host cell recognition. The active site of 7JSU protein is constituted by His41 and Cys145 of the catalytic dyad, and important residues like Glu166, Phe140, and His163 that play a part in substrate recognition. The S1 pocket is hydrophilic, while hydrophobic interactions are facilitated by S2 and S4 pockets. The 7DE1 protein binding pocket, the RNA-binding domain (RBD), with the RNA-binding groove and the most critical residues (Arg92, Tyr109, and Phe115) responsible for stabilizing viral genome packaging. Docking is to search for compounds that interfere with RNA-protein interactions.” [Page 23-24, Section 3.3]

**Comment 4:** *[*The authors should elaborate more on the ligand-target interaction patterns. Ligand-residue interactions should be annotated in terms of both the bond distances and angles. Especially for Hydrogen bonding, this type of compound-protein polar interaction should be presented within hydrogen bond distances as well as bond angles since hydrogen bond depend on both. Authors should mention the Hydrogen bond angles as well as their distances, since the strength of hydrogen bonding is based on both parameters in a way to ensure the adequacy of optimum hydrogen bonding.*]*

**Response:** We appreciate the reviewer’s insightful suggestion to provide a more detailed analysis of ligand-target interaction patterns, particularly regarding hydrogen bonding distances and angles. We have revised our manuscript in the light of reviewer’s comments as follow:

“Fig 5 represents the 2D interaction view of optimal pose of five top-ranked drug compounds interacting with the target proteins *7JSU, 7LM9* and *7DE1* proteins. The best binding affinities for these drug compounds range from -19.7 to -12.6 kcal/mol. The precise orientation and conformation of the ligand molecule that results in the lowest binding affinity with the target protease is referred to as the "optimal pose." The ideal ligand posture with respect to the target is indicated by the binding energy value that is the highest negative. Van Der Waals, conventional hydrogen bonds, carbon hydrogen bonds, Pi-cations, Pi-sulfur, Pi-Pi T-shaped, unfavorable donor-donor, alkyl, and Pi-alkyl interactions are all depicted in 2D view of the protein-ligand interaction. The overall stability of protein-ligand binding is significantly influenced by these many kinds of interactions.

**Fig 5.** 2D interaction view of top ranked ligands with 7JSU, 7LM9 and 7DE1 Proteins.

Among many of non-covalent forces, hydrophobic interaction and hydrogen bonding are particularly important in stabilizing the ligand inside the active site in protein–ligand interactions. Nonpolar regions of the ligand and protein avoid water, hence pushing them together and causing hydrophobic interactions. By helping good van der Waals forces and lowering the entropic cost of solvation, these interactions greatly affect the binding affinity. Conversely, hydrogen bonds provide directionality and specificity, typically serving as major factors in ligand identification. Typical hydrogen bonds include an electronegative donor and acceptor with bond distances ranging from 2.5 Å to 3.5 Å and bond angles preferably above 120°, both of which are essential for maximum binding strength. The detail of hydrophobic interaction and H-bond is given in the supplementary file S1.” [Page 27-28, Section 3.3]

**Comment 5:** *[*It is advised that the authors provided a MM_PB(GB)SA free binding energy calculations with dissected energy terms (ΔGelectrostatic, ΔGvdw, ΔGsolvation,…..) to highlight the dominant potential for guiding further compound optimization and development. Identifying the hotspot residues through binding energy contribution is also advised. FastDRH webserver is feasible to perform such approach (<http://cadd.zju.edu.cn/fastdrh/)>.*]*

**Response:** Thanks to the reviewer’s suggestion to perform **MM-PB(GB)SA free binding energy calculations;** however, our study is based on **AutoDock Vina,** which does not inherently support such calculations. Vina provides **binding affinity scores (ΔG in kcal/mol)** based on a force-field-based scoring function. To address the reviewer's concern, we have tried to use (<http://cadd.zju.edu.cn/fastdrh/)> but this server always give the error of failed request to status code 502.

**Comment 6:** *[*Based on the study results, what are the take-away messages. Authors are advised to highlight the suggested structural modifications that would improve the compound’s affinity based on the in silico findings. These insights would be beneficial for guiding future lead optimization and development*]*

**Response:** Thank you for your valuable suggestion. We have revised our manuscript as follow:

“Strong hydrogen bonding and hydrophobic interactions with important active site residues supported the favorable binding affinities of the topranked ligands (such as ZINC085432544, ZINC003873365, ZINC085536956, ZINC261494640 and ZINC008214470) across several SARS-CoV-2 target proteins as discovered in silico. The interaction analysis indicates that including more polar functional groups, especially those able to form directional hydrogen bonds, could still increase binding specificity and strength. Moreover, improving the packaging within the binding pocket would entail maximizing the spatial orientation of hydrophobic groups. These structural insights offer a useful foundation for prospective compound optimization work, thus directing the design of more powerful and selective antiviral candidates.” [Page 28, Section 3.3]

**Reviewer 2 Comments and Responses:**

**Comment 1:** *[*Describe the cross-validation methodology and machine learning model hyperparameter selection*]*

**Response:** Thank you for your valuable feedback. Cross Validation Methodology is described in the manuscript. [Page 16, Section 2.2.3]

Machine Learning Hyperparameter selection is described in Table 2

**Table 2.** Details of hyper-parameters setting of predictive models. [Page 16, Section 2.2.3]

**Comment 2:** *[*Explain the superior performance of Decision Tree Regression (DTR) over alternative models.*]*
**Response:** Thank you for your valuable feedback. We revised our manuscript as follow:

“Decision Tree Regression (DTR) is more effective than models like GBR, XBGR, KNNR, ETR and MLPR. This is because it is better in the detection of non-linear correlations between features/independent variables and binding affinities. It is not subject to any distributional assumption on the data compared to parametric models, so it is more flexible. It chooses relevant features, minimizing over-fitting and maximizing efficiency, as opposed to KNNR, which does not perform well with high-dimensional data. Furthermore, DTR is less sensitive to outliers and computationally more efficient compared to ensemble-based approaches such as GBR, ETR and XGBR. All these strengths position DTR as a promising contender for drug repurposing binding affinity prediction.” [Page 15, Section 2.2.3]

**Comment 3:** *[*Provide a list of the top-ranked ligands along with their characteristics in an additional file.*]*

**Response:** Thank you for your valuable feedback. We agree to provide a list of the top-ranked ligands in the supplementary file S2.

**Comment 4:** *[*Offer in vivo or in vitro tests as a means of verification*]*

**Response:** Thank you for your valuable feedback. We have described in our manuscript at [Page 34, Section 4]

**Comment 5:** *[*Indicate whether any of the medication candidates that were found had antiviral properties.*]*

**Response:** Thank you to this valuable suggestion. We have incorporated the following text in the revised manuscript:

“The top-ranked identified ligands (ZINC003873365, ZINC085432544, ZINC085536956, ZINC008214470, and ZINC261494640) are mostly anticancer drugs. Although their antiviral effects have not been directly determined, their high affinities in binding to SARS-CoV-2 proteins (Spike, 3CLpro, and Nucleocapsid) indicate repurposing potentials. Most anticancer drugs display broad-spectrum activity, such as antiviral, based on mechanisms including kinase inhibition, immune modulation, or apoptosis induction. Further in vitro and in vivo work is required to establish whether the compounds have direct antiviral activity against SARS-CoV-2.” [Page 33, Section 3.4]

**Comment 6:** *[*Verify that every figure has the appropriate label and has all relevant information (such as R2 values in regression graphs).*]*

**Response:** Thank you for your valuable feedback. We have verified the label of every figure.

**Reviewer 3 Comments and Responses:**

**Comment 1:** *[*The title should be more descriptive or comprehensive title that fully reflects the scope and depth of your study*]*

**Response:** Thank you for your valuable feedback. We have changed the title as advised in the revised manuscript as follow:

In-silico study of approved drugs as potential inhibitors against **3CLpro and other viral proteins** of Covid-19 [Page 1]

**Comment 2:** *[*The abstract is well-written overall, but by tightening up some sentences and providing a bit more detail on the methodology and results, you can enhance its clarity and impact.*]*

**Response:** Thank you for your suggestion. To improve the clarity and impact, we revised the abstract in the revised manuscript as follow:

“The global pandemic, due to the emergence of COVID-19, has created a public health crisis. It has a huge morbidity rate that was never comprehended in the recent decades. Despite numerous efforts, potent antiviral drugs are lacking. Repurposing of drugs presents a low-cost and rapid solution for finding new drugs by exploiting known drugs. In this study, we employed an integrated In-Silico approach using molecular docking and machine learning regression models to explore the potential inhibitors against key proteins of SARS-CoV-2. A library of 5903 drugs from the ZINC database was retrieved and screened against three crucial viral targets: *Spike* glycoprotein *(7LM9)*, *main protease 3CLpro (7JSU)*, and *Nucleocapsid protein (7DE1).* Binding affinities were predicted by using molecular docking, and subsequent predictive regression models, Decision Tree Regression (DTR), Gradient Boosting, XGBoost, Extra Trees, KNNR, and MLP, were constructed employing MACCS molecular fingerprints. Among them, the DTR model had better predictive performance, as indicated by the highest R² and lowest RMSE. The highest ranked compounds possessed good binding affinities (−12.6 to −19.7 kcal/mol) and favorable pharmacokinetics. Importantly, five novel candidate compounds, namely ZINC003873365, ZINC085432544, ZINC008214470, ZINC085536956, and ZINC261494640, had multi-target potential and optimal binding interaction. This computational analysis yields useful information for lead prioritization and sets the stage for additional in vitro and in vivo confirmation of these drug candidates to combat COVID-19.” [Page 1-2]

**Comment 3:** *[*Try to improve your introduction and compare your results to others like (<https://doi.org/10.2147/DDDT.S354841)>.*]*

**Response:** Thank you for your suggestion. We have incorporated the following text in the revised manuscript:

“In a study [14], a number of antiviral drug classes and recently authorized oral medicines, including molnupiravir and PF-07321332, have demonstrated effectiveness against SARS-CoV-2 by inhibiting viral replication.” [Page 4]

**Comment 4:** *[*Write in silico, in vitro, and in vivo words in italic*]*

**Response:** Thank you for your valuable feedback. We have done in the revised manuscript.

**Comment 5:** *[*Overall, the article is well-structured and easy to follow. The introduction provides a clear context for the research, and the progression through various sections is logical and cohesive. However, some sections could benefit from clearer transitions. For instance, the section on in silico approaches could have more detailed explanations about the specific algorithms and software used in the studies, which would provide readers with a deeper understanding of the methodologies.*]*

**Response:** Thank you for your valuable feedback. Mostly software and algorithm used are already mentioned in the manuscript. We have mentioned remaining tools used in the revised manuscript as follow:

These calculations are got by using the webserver (<https://proteins.plus/>). [Page 24]

**Comment 6:** *[*Molecular dynamics simulation (for at least 100 ns) for the most active five complexes, compared to the co-crystallized inhibitor of each target receptor should be per formed to confirm the docking results.*]*

**Response:** Thank you for your insightful suggestion. While our current study focuses on molecular docking to predict binding affinity, we plan to include MD simulations in future work to complement these results and better capture the dynamic nature of protein-ligand interactions.

**Comment 7:** *[*Some tables (like Tables 7, 8, and 9) should be moved to the Supplementary Data.*]*

**Response:** Thank you for your valuable feedback. We agreed and moved the Table 7 in the supplementary file. Table 8 shows the physiochemical properties of selected drug compounds and we have detailed analysis of it. So the reader may feel the difficulty if it moves in the supplementary data.

**Comment 8:** *[*A graphical abstract is recommended*]*

**Response:** We appreciate the suggestion regarding the inclusion of a graphical abstract. However, in accordance with the submission guidelines of PLOS ONE, graphical abstracts are not part of the journal’s publication policy. Therefore, we have adhered to the journal's standards by focusing on a clear and comprehensive written presentation of our study.

**Comment 9:** *[*The article fails to adequately discuss the importance of experimental validation following in silico predictions. While in silico techniques are powerful, the review does not highlight how real-world testing (e.g., in vitro or in vivo experiments) is essential to confirm the efficacy and safety of the identified inhibitors. The reliance on computational data alone can be problematic, and without a proper discussion of the need for follow-up experiments, the paper might give the impression that in silico studies are sufficient on their own.*]*

**Response:** We thank the reviewer for raising this important point. We agree that in silico predictions must be supported by experimental validation to ensure clinical relevance. As noted in the **Conclusion** section of our manuscript, we have explicitly acknowledged this limitation and emphasized the necessity for follow-up **in vitro** and **in vivo** studies to validate the efficacy and safety of the identified inhibitors. We also stated our intention to pursue such validation in future work.

“Follow-up research, including *in vitro* and *in vivo* studies, is necessary to confirm their effectiveness. Moreover, exploring the possible synergistic effects of these compounds in combination with other treatments could yield valuable insights. Our methodology could also be applied to other viral diseases and conditions beyond virology, making drug repurposing a promising strategy. This study highlights the significance of utilizing computational tools to expedite the drug discovery process, particularly during emerging pandemics and urgent health challenges. The scope of this study is limited to *in-silico*. However, in the future, we intend to validate these computational findings through experimental studies, including *in vitro* assays to assess binding affinity and inhibitory activity, as well as *in vivo* studies to evaluate pharmacokinetics.”[Page 34, Section 4]

**Comment 10:** [Improve the resolution of the 2D structures in table 7, so that the chemical structure, such as bond angles and atom labels are clear even when printed or zoomed in.*]*

**Response:** Thank you for your valuable feedback. We have improved the resolution of the 2D structures in Table 7.

**Comment 11:** *[*There are some grammatical and typographical error, please revise it.*]*

**Response:** Thank you for your valuable feedback. We have revised the manuscript to avoid the grammatical and typographical error.
